# Effect of Prophylactic Levosimendan on All-Cause Mortality in Pediatric Patients Undergoing Cardiac Surgery—An Updated Systematic Review and Meta-Analysis

**DOI:** 10.3389/fped.2020.00456

**Published:** 2020-08-14

**Authors:** Hongbai Wang, Qipeng Luo, Yinan Li, Liang Zhang, Xie Wu, Fuxia Yan

**Affiliations:** ^1^Department of Anesthesiology, Fuwai Hospital, Chinese Academy of Medical Sciences and Peking Union Medical College, Beijing, China; ^2^Department of Anesthesiology, Chongqing Traditional Chinese Medicine Hospital, Chongqing, China

**Keywords:** low cardiac output syndrome, levosimendan, pediatrics, cardiac surgery, mortality

## Abstract

**Background:** Levosimendan, a calcium sensitizer, enhances the myocardial function by generating more energy-efficient myocardial contractility than that achieved through adrenergic stimulation with catecholamines. We conducted this meta-analysis to primarily investigate the effects of levosimendan on all-cause mortality in pediatric patients undergoing cardiac surgery under cardiopulmonary bypass.

**Methods:** The databases of Pubmed, Embase, and Cochrane Library were searched till 21st March 2020. The eligible criteria were participants with age<18 year and undergoing cardiac surgery for congenital heart disease (CHD), and studies of comparison between levosimendan and placebo or other inotropes. Stata version 12.0 was used to perform statistical analyses.

**Results:** Six randomized controlled trials (RCTs) and 1 case–control trial (CCT) including 436 patients were included. The results showed that levosimendan did not significantly decrease all-cause mortality compared with control drugs (and placebo) in children undergoing cardiac surgery (*P* = 0.403). Perioperative prophylactic levosimendan administration strikingly decreased the low cardiac output syndrome (LCOS) incidence (*P* = 0.016) but did not significantly reduce acute kidney injury (AKI) incidence (*P* = 0.251) and shorten mechanical ventilation and ICU stay time compared with other inotropes and placebo by analyzing the included literatures [mechanical ventilation (or intubation) time: *P* = 0.188; ICU stay time: *P* = 0.620].

**Conclusions:** Compared with other inotropes and placebo, perioperative prophylactic administration of levosimendan did not decrease the rates of mortality and AKI and shorten the time of mechanical ventilation (or intubation) and ICU stay but demonstrated a significant reduction in LCOS incidence after corrective surgery in pediatric patients for CHD. Due to limited number of included studies, the current data were insufficient to make the conclusions.

## Introduction

Low cardiac output syndrome (LCOS) refers to the clinical manifestation of mismatched oxygen supply and demand due to cardiovascular dysfunction following cardiac surgery (1). There are no clear diagnostic criteria of LCOS in children, especially infants and neonates. Some parameters for LCOS provided by several authors in children include (1) elevated blood lactate or rapid increase in blood lactate; (2) decreased central venous oxygen saturation; (3) increase in arterial to central venous oxygen saturation difference; (4) decreased urine output; (5) increased peripheral skin temperature to core body temperature difference; (6) echocardiographic Doppler-derived low cardiac index; and (7) high inotrope requirement (2). LCOS often occurs during 9–12 h after cardiopulmonary bypass (CPB) (3). The incidence of LCOS is nearly 10% (9.98%) in children (0–18 years old) after corrective surgery for congenital heart disease (CHD), while those in neonates and infants were as high as 25–65% (4, 5). The development of LCOS is highly associated with acute kidney injury (AKI), prolonged time of mechanical ventilation and ICU stay, and even higher mortality (6–10).

Some inotropic agents have been widely used in clinical practice to prevent and treat LCOS. Catecholamines (epinephrine, norepinephrine, dopamine, and dobutamine) and phosphodiesterase inhibitor (milrinone) are the traditional prophylactic and therapeutic medications. However, these drugs are associated with considerable side effects (11, 12). Levosimendan is a novel inotropic drug that enhances myocardial contractility through increasing the sensitivity of calcium ion to cardiomyocytes (13). In addition, levosimendan also has the pharmacological feature of dilatation of blood vessels (systemic, pulmonary, and coronary) due to its role of K^+^ efflux, thereby decreasing cardiac preload and afterload (14). Therefore, levosimendan elevates cardiac contractility, meanwhile it does not increase cardiac oxygen consumption. Besides, levosimendan has a pharmacological feature of myocardial preservation as well (15). The mechanisms involved improvement of myocardial tissue perfusion (coronary blood flow) and prevention of mitochondrial calcium overload via an increase in potassium influx induced by levosimendan (15). Therefore, levosimendan has a theoretical advantage in improving post-operative cardiac function and reducing post-operative complications and mortality in pediatric patients undergoing cardiac surgery. We designed this meta-analysis to primarily observe the effect of perioperative prophylactic levosimendan administration on all-cause mortality in pediatric patients following cardiac surgery under CPB.

## Methods

This systematic review and meta-analysis was conducted according to the guidelines of the 2009 PRISMA (Preferred Reporting Items for Systematic reviews and Meta-analyses) [Supplementary Table 1; (16)].

### Search Strategy and Study Selection

We searched the databases including Pubmed, Embase, and Cochrane Library through PICOS (patients, intervention, control, outcome, and study design) strategy by the time to 21st March 2020. The entry words included “infant” or “newborn” or “child” or “children” or “pediatrics” or “neonate” and “simendan” or “levosimendan” and “thoracic surgery” or “surgery, thoracic” or “surgery, cardiac” or “cardiac surgery” or “heart surgery” and “mortality” or “mortalities” or “case fatality rate” or “rate, case fatality” or “rates, fatality” or “death rate” or “rate, death” or “rates death” or “mortality rates,” and the search scope was “all fields.” Because all studies about the effect of levosimendan vs. placebo or other inotropic drugs on mortality in pediatric patients were eligible in this meta-analysis, we did not confine the search words of control drugs and study design. The inclusion criteria included (1) participants with age<18 years and (2) management with prophylactic levosimendan and placebo or other inotropic agents. The exclusion criteria included (1) participants with age ≥18 years; (2) review or meta-analysis; (3) basic research; (4) article published as abstract, letter, case report, editorial, note, method, or protocol; and (5) article presented in non-English language.

### Data Analysis

The primary outcome was all-cause mortality after cardiac surgery. The secondary outcomes included the incidence of LCOS and AKI, and time of mechanical ventilation (or with tracheal tube) and ICU stay post-operatively.

Hongbai Wang and Yinan Li independently reviewed the titles, abstracts, or both; summarized the data of the included literatures; and extracted the following information: (1) authors; (2) publication year; (3) number of the total participants in each study; (4) age range of all the participants; (5) country of publication; (6) time of levosimendan, or other drugs administration; (7) infusion speed of levosimendan or other sedatives; and (8) number of patients suffering death or acute kidney injury (AKI), and time of mechanical ventilation (or intubation) and ICU stay following cardiac surgery. In addition, we also collected the mortality categories and scores of the Society of Thoracic Surgeons-European Association for Cardiothoracic Surgery (STS-EACTS) (17) or risk categories according to the Risk Adjustment for Congenital Heart Surgery (RACHS) (18). Liang Zhang, Fuxia Yan, and Xie Wu were responsible for checking the accuracy of data.

Hongbai Wang and Liang Zhang independently evaluated the quality of included articles. The risk of bias of randomized controlled trials (RCTs) were assessed by the Cochrane Collaboration Risk of Bias Assessment tool, which included seven items, i.e., random sequence generation, allocation concealment, blinding of participants and personnel, blinding of outcome assessment, incomplete outcome data, selective reporting, and others (bias due to vested financial interest, and academic bias). If a trial had one or more of the items to be judged as high or unclear risk of bias, this trial was classified as having high risk (19). The bias risk of case–control trials (CCTs) were assessed by the Newcastle–Otawa Quality Assessment Scale (NOS) which comprised three domains: selection, comparability, and outcome for cohort studies. There are four stars in the selection domain, two stars in the comparability domain, and three stars in the exposure domain. Studies with cumulative 7 stars or more are considered to be of high quality, 6 stars to be of moderate quality, and <6 stars to be of low quality [Supplementary Table 2; (20)]. If the two authors obtained the different assessment results, they consulted the third or fuorth one. Eventually, we reached consensus.

Stata version 12.0 (Stata Corp, College Station, TX, USA) was used to perform statistical analyses. The values of *I*^2^ and the Mantel–Haenszel chi-square test (*P*-value for heterogeneity) were used to evaluate the heterogeneity of included studies. Moreover, the values of *I*^2^ <40%, 40–60%, and >60% represented low, moderate, and high heterogeneity, respectively (21). If *I*^2^ >50% or a *P*-value for heterogeneity<0.1 was identified, the method of random-effect model analysis was applied to analyze the data. Contrarily, if *I*^2^ <50% or a *P-*value for heterogeneity≥0.1 was presented, the method of the fixed-effect model was used (22). The dichotomous outcomes were reported as relative risk (RR) with 95% confidence interval (CI). Because the different time units (hours and days) were presented in mechanical ventilation (or intubation) time and ICU stay time, the two continuous outcomes were analyzed as standard mean difference (SMD) with 95% CI (23). Subgroup analyses were conducted for primary outcome according to study designs, control drugs, onset time of study drugs, and duration of study drug infusion. The *P*-value with two-sided tests for effect <0.05 was considered significant differences.

## Results

### Study Location and Selection

**Figure 1:**
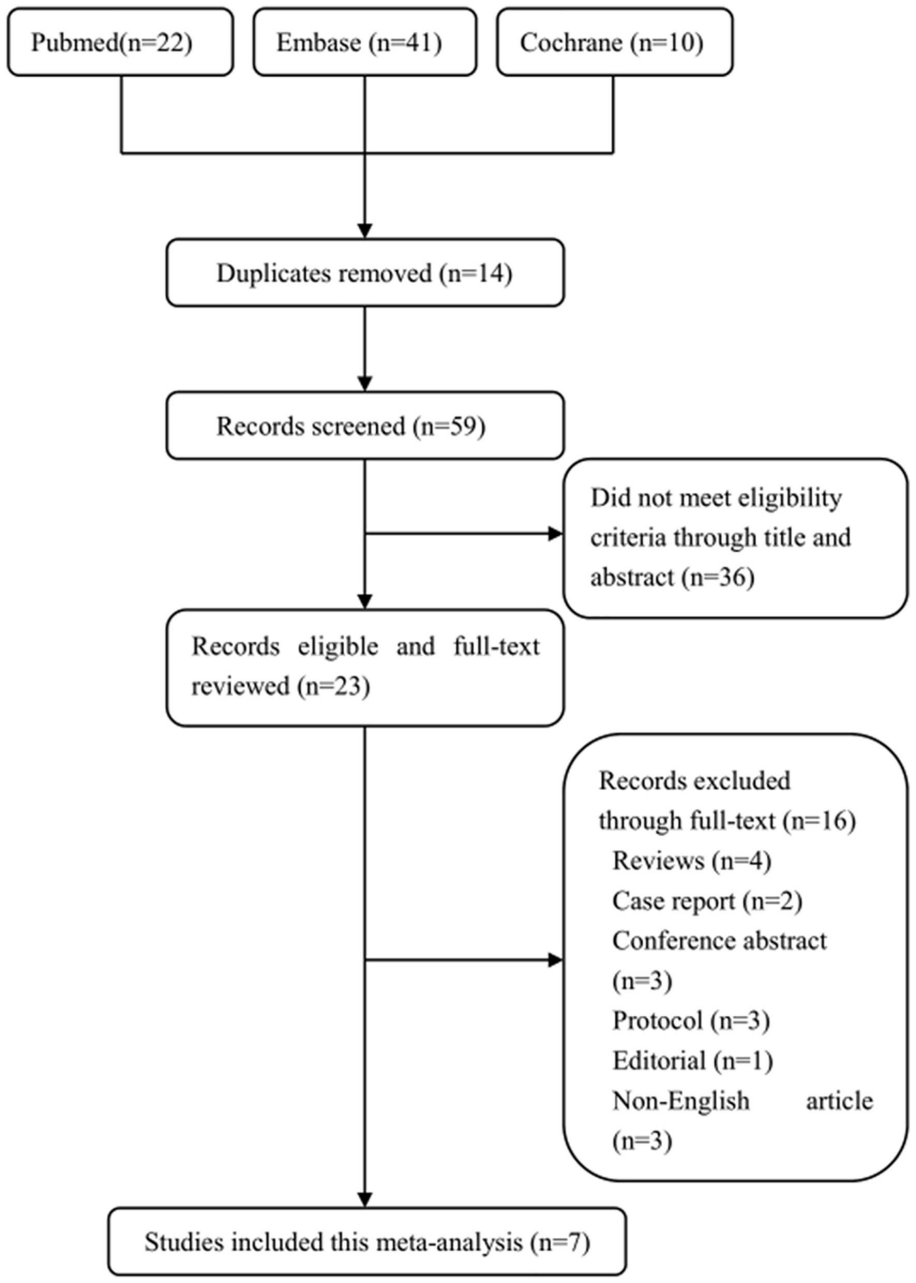
The screening process of the eligible trials.

The screening process of the eligible studies is shown in Figure 1. We obtained 22 literatures in Pubmed, 41 in Embase, and 10 in Cochrane Library according to inclusion criteria. Fourteen literatures were removed due to duplicates. Thirty-six literatures were excluded because they did not meet the eligible criteria by browsing the titles and abstracts, and 16 literatures were removed by browsing the full text. Eventually, 7 trials including 436 patients were indentified through our search strategy [Figure 1; (24–30)].

### Characteristics of Included Trials

The basic information of all included trials is shown in Table 1. Four trials (24, 26–28) were conducted in European countries, and 3 trials (25, 29, 30) in Asian countries. All patients included in the 7 articles were under 5 years old. One trial (26) selected the pediatric patients younger than 1 year, and 2 trials (27, 28) younger than 30 d. Six literatures (24–28, 30) including 396 patients were RCTs, and 1 literature (29) including 40 patients was CCT. There were 3 articles (24, 26, 27) comparing levosimendan with milrinone, 1 article (25) with dobutamine, 1 article (30) with placebo, and 2 articles (28, 29) with the standard inotropic management. The loading dose was administrated in 1 trial (25). The intervention was started before surgery in 1 trial (27), during surgery in 4 trials (24–26, 28), and after surgery in 2 trials (29, 30). The infusion duration of study drugs was 24 h after surgery in 3 trials (25, 26, 29), 48 h after starting infusion in 3 trials (24, 27, 30), and 72 h after starting infusion in 1 trial (28). The number of patients suffering death, LCOS and AKI, and the duration of mechanical ventilation (or intubation) and ICU stay are shown in Table 2. There were no significant differences in mortality risk between groups of levosimendan and control in included trials according to STS-EACTS [Supplementary Tables 3, 4; (24–29)] or RACHS (30).

**Table 1:** The basic information of all included trials.

**Author/publication year (method)**	**Center/country**	**Number of patients**	**Age**	**Infusion time**	**Levosimendan**	**Control drug**
Momeni et al. (24) (RCT)	Single/Belgium	36	7–977 d	**Beginning:** the onset of CPB**Duration:** a maximum of 48 h	**Dose:** 0.05–0.1 μg/kg/min	**Milrinone:** 0.4–0.8 μg/kg/min
Ebade et al. (25) (RCT)	Single/Egypt	50	7–38 m	**Beginning:** immediately after declamping of the aorta**Duration:** 24 h from the time of admission to the ICU	**Loading:** 15 μg/kg over a 10 min**Continuous rate:** 0.1–0.2 μg/kg/min	**Dobutamine:** 4–10 μg/kg/min
Lechner et al. (26) (RCT)	Single/Austria	40	<1 y	**Beginning:** at the time of weaning from CPB**Duration:** the first post-operative 24 h	**Dose:** 0.1 μg/kg/min	**Milrinone:** 0.5 μg/kg/min
Pellicer et al. (27) (RCT)	Single/Spain	20	<30 d	**Beginning:** before surgery**Duration:** 48 h after starting infusion	**Dose:** 0.1–0.2 μg/kg/min	**Milrinone:** 0.5–1.0 μg/kg/min
Ricci et al. (28) (RCT)	Single/Italy	63	<30 d	**Beginning:** at the time of weaning from CPB**Duration:** 72 h	**Dose:** 0.1 μg/kg/min	The standard inotropic management
Wang et al. (29) (CCT)	Single/China	40	3.0–22.0 m	**Beginning:** after surgery **Duration:** 24 h	**Dose:** 0.1–0.2 μg/kg/min	Not levosimendan
Wang et al. (30) (RCT)	Single/China	187	≤48 m	**Beginning:** after surgery **Duration:** 48 h	**Dose:** 0.05 μg/kg/min	Placebo

**Table 2:** The primary and secondary outcomes of included trials.

**Study**	**Number of patients**		**All-cause mortality**		**LCOS**		**AKI**		**Mechanical ventilation (or intubation) duration**		**ICU stay**	
	**Levo**	**Control**	**Levo**	**Control**	**Levo**	**Control**	**Levo**	**Control**	**Levo**	**Control**	**Levo**	**Control**
Momeni et al. (24)	18	18	1	1	NA	NA	0	0	77 (2–167) (h)	34 (3–237) (h)	7 (2–15) (d)	3 (2–20) (d)
Ebade et al. (25)	25	25	0	0	NA	NA	NA	NA	6 ± 1.9 (h)	7 ± 1.6 (h)	47.3 ± 2.9 (h)	49.3 ± 3.4 (h)
Lechner et al. (26)	19	20	0	0	NA	NA	NA	NA	4 (3–6) (d)	4 (2–8) (d)	6 (5–8) (d)	6.5 (5–11.5) (d)
Pellicer et al. (27)	11	9	0	1	3	3	NA	NA	NA	NA	NA	NA
Ricci et al. (28)	32	31	1	3	12	19	0	0	NA	NA	NA	NA
Wang et al. (29)	20	20	2	1	NA	NA	5	8	146.0 (76.5–888.0) (h)	27.0 (11.0–75.0) (h)	10.5 (7.3–39.3) (d)	4.0 (2.0–10.0) (d)
Wang et al. (30)	94	93	3	4	10	18	1	2	47.5 (21.4–96.0) (h)	39.5 (18.0–97.3) (h)	114.5 (72.38–189) (h)	118 (69–200.25) (h)

### Bias Risk Assessment

Bias risk of 6 RCTs was assessed by the Cochrane Collaboration Risk of Bias Assessment tool. Random sequence generation was assessed as a low risk of bias in 6 studies (100%), allocation concealment in 5 studies (83%), blinding of participants in 4 studies (67%), blinding of outcome assessment in 6 studies (100%), incomplete outcome data in 4 studies (67%), selective outcome reporting in 6 studies (100%), and other bias in 5 studies (Supplementary Figures 1, 2). The CCT study obtained 7 stars through NOS. One RCT (25) and CCT (29) were assessed to be of high quality.

### The Primary Outcome

**Figure 2:**
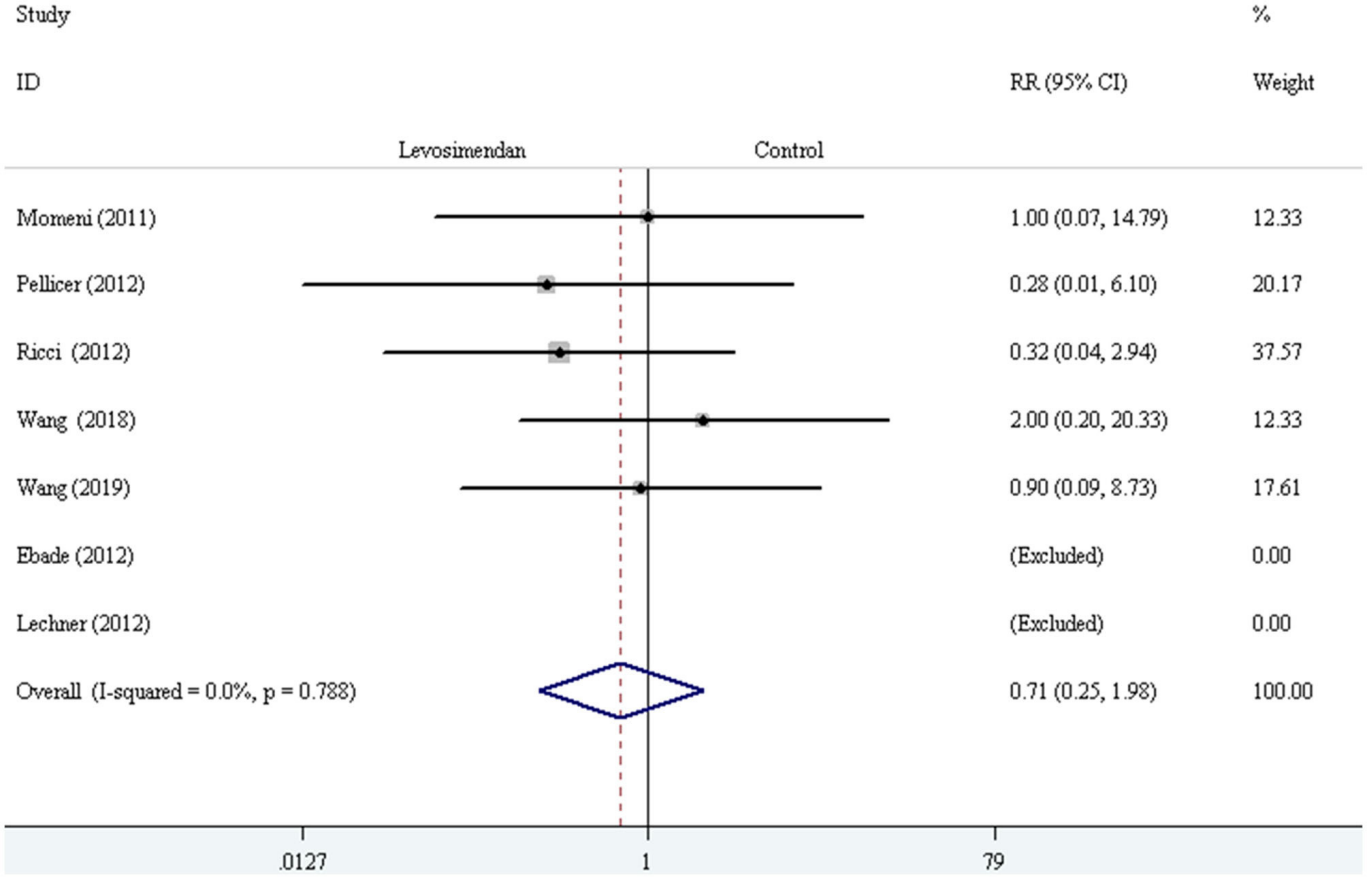
The comparison of all-cause mortality between levosimendan and control drugs or placebo.

The fixed-effect model with RR was selected to evaluate the primary outcome, and the pooled result did not demonstrate significant difference in all-cause mortality compared levosimendan with control drugs (and placebo) [RR = 0.71, 95% CI (0.25, 1.98), *I*^2^ =0, *P* for effect = 0.507] (Figure 2).

**Figure 3:**
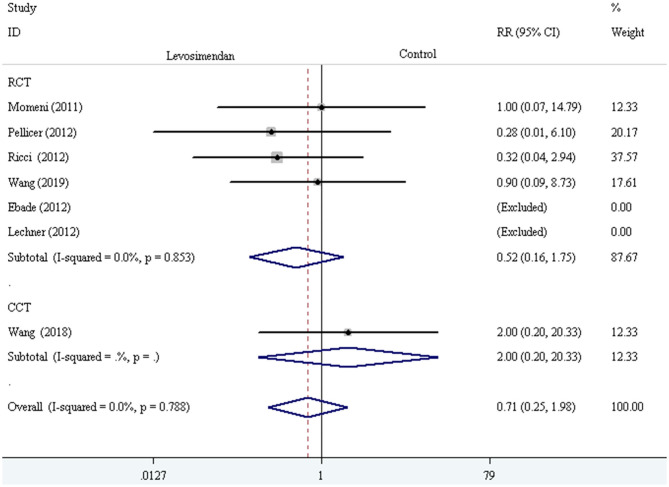
The subgroup analysis of all-cause mortality according to study designs.

**Figure 4:**
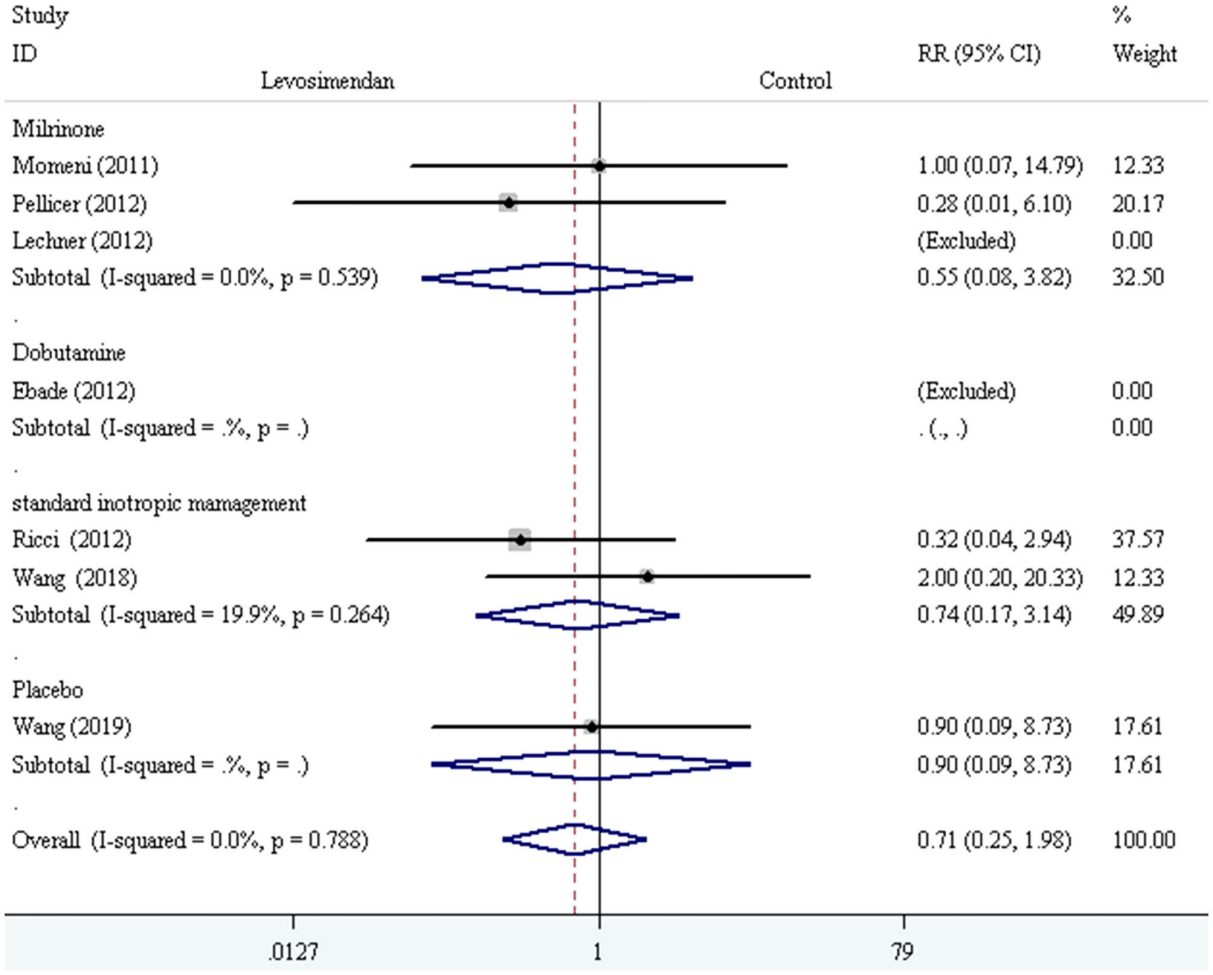
The subgroup analysis of all-cause mortality according to control drugs.

**Figure 5:**
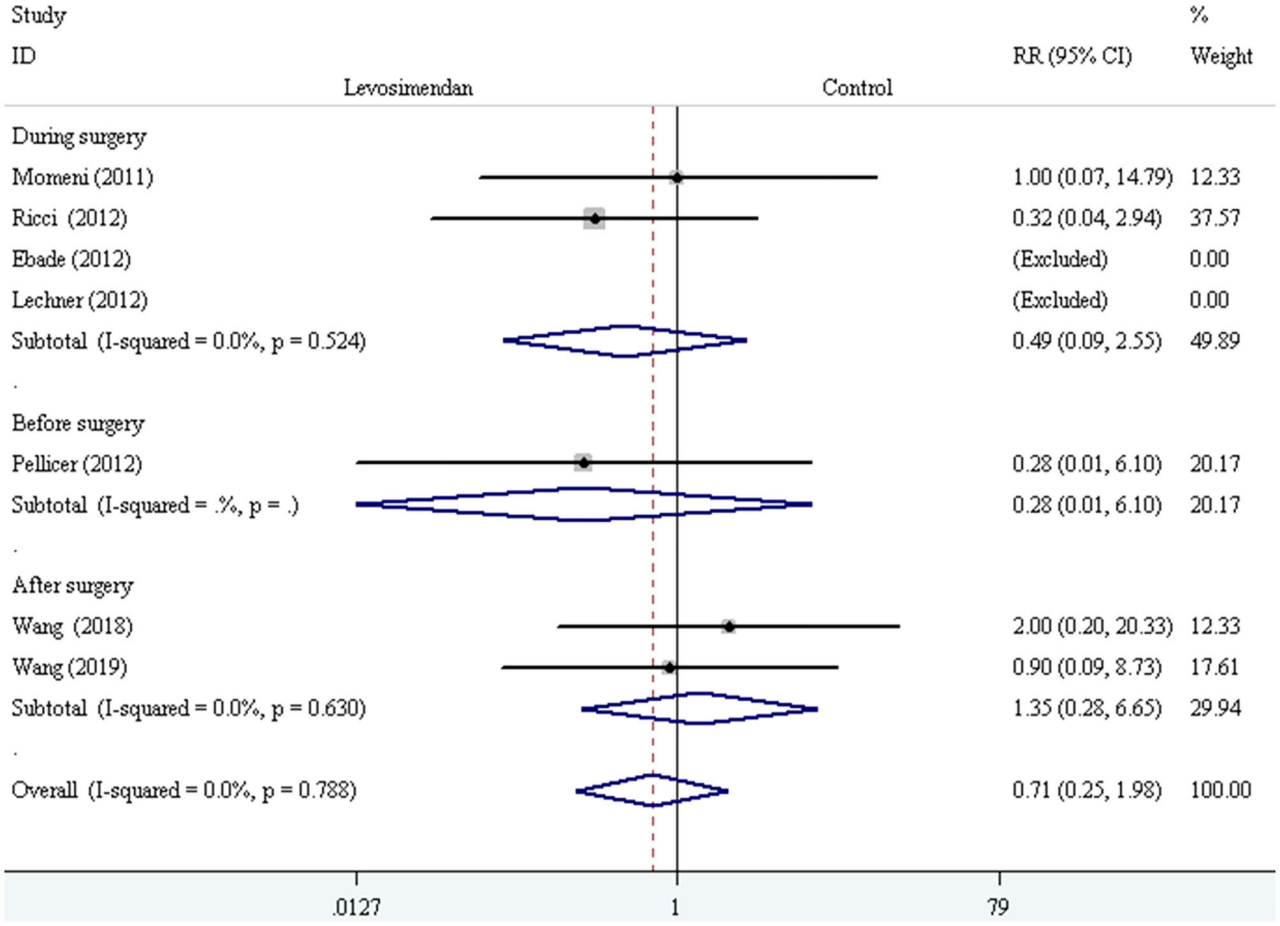
The subgroup analysis of all-cause mortality according to time of drug infusion onset.

**Figure 6:**
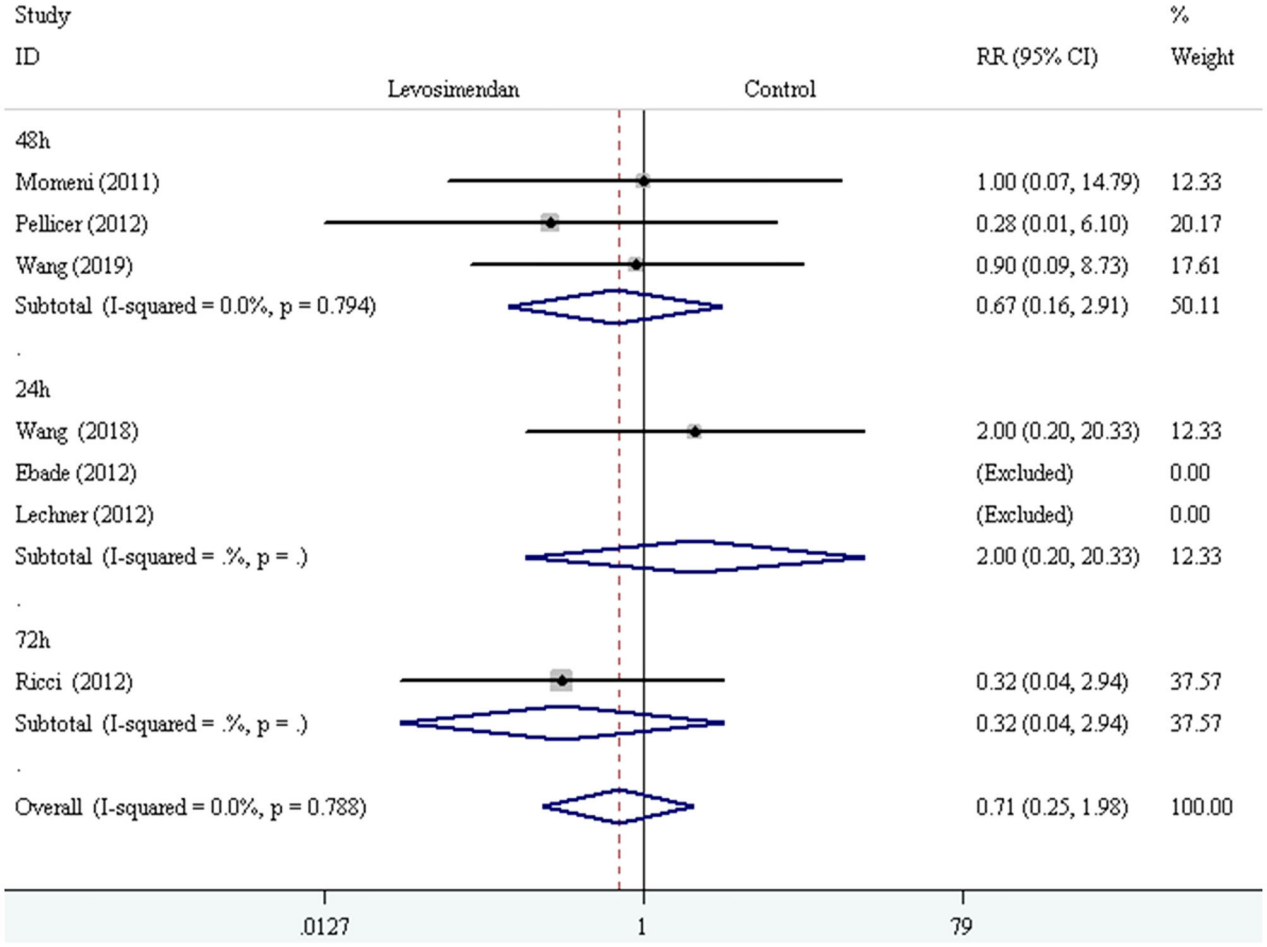
The subgroup analysis of all-cause mortality according to duration of study drug infusion.

We conducted the subgroup analyses according to study designs, control drugs, time of study drug infusion onset, and duration of study drug infusion. There was no significant difference in all-cause mortality between groups of levosimendan and control according to study designs [RCTs: RR = 0.52, 95% CI (0.16, 1.75), *P* for effect = 0.293; CCT: RR = 2.00, 95% CI (0.20, 20.33), *P* for effect = 0.558] (Figure 3), control drugs [milrinone: RR = 0.55, 95% CI (0.08, 3.82), *P* for effect = 0.547; standard inotropic management: RR = 0.74, 95% CI (0.17, 3.14), *P* for effect = 0.680; placebo: RR = 0.90, 95% CI (0.09, 8.73), *P* for effect = 0.928] (Figure 4), time of drug infusion onset [before surgery: RR = 0.28, 95% CI (0.01, 6.10), *P* for effect = 0.416; during surgery: RR = 0.49, 95% CI (0.09, 2.55), *P* for effect = 0.397; after surgery: RR = 1.35, 95% CI (0.28, 6.65), *P* for effect = 0.710] (Figure 5), and duration of study drug infusion [24 h after surgery: RR = 2.00, 95% CI (0.20, 20.33), *P* for effect = 0.558; 48 h after starting infusion: RR = 0.67, 95% CI (0.16, 2.91), *P* for effect = 0.597; 72 h after starting infusion: RR = 0.32, 95% CI (0.04, 2.94), *P* for effect = 0.316] (Figure 6).

### The Secondary Outcomes

**Figure 7:**
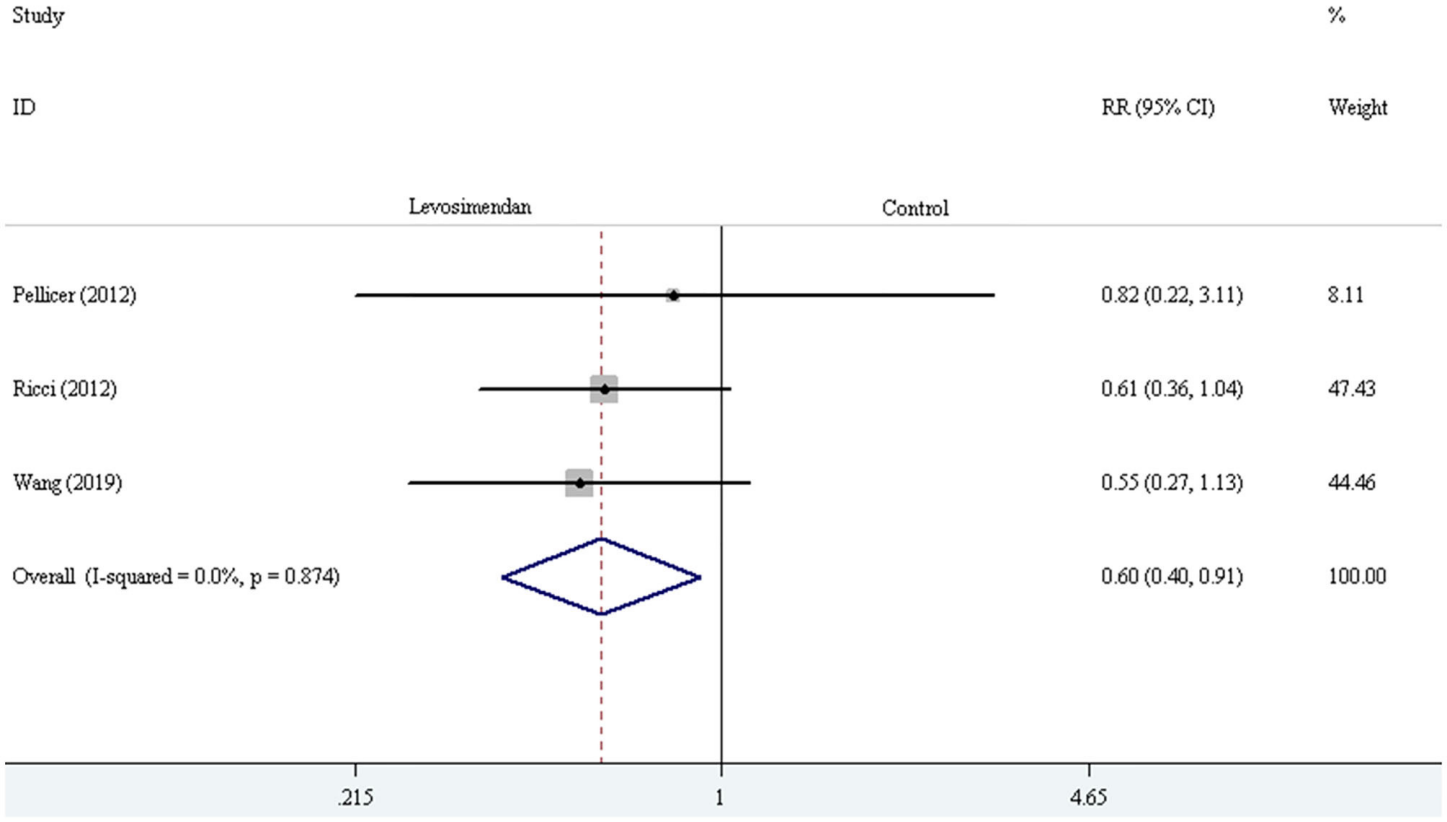
The comparison of LCOS incidence between levosimendan and control drugs or placebo.

**Figure 8:**
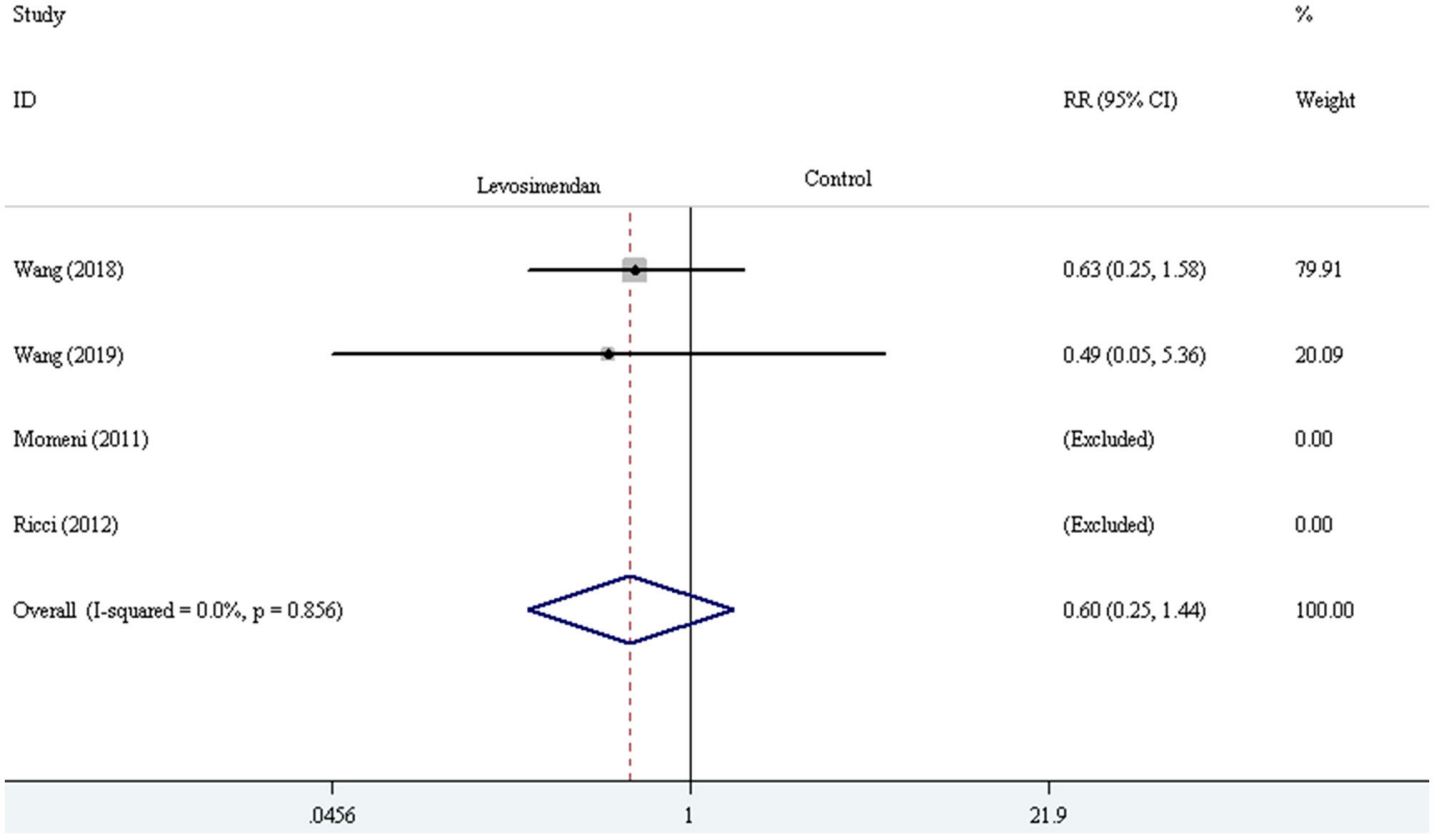
The comparison of AKI incidence between levosimendan and control drugs or placebo.

Three trials demonstrated the incidence of LCOS (27, 28, 30). The pooled result showed striking difference in LCOS incidence [RR = 0.60, 95% CI (0.40, 0.91), *I*^2^ = 0, *P* for effect = 0.016] (Figure 7). Four trials reported the incidence of AKI (24, 28–30). The pooled result did not demonstrate a significant difference in AKI incidence comparing levosimendan with control drugs (and placebo) through the method of the fixed-effect model with RR [RR = 0.60, 95% CI (0.25, 1.44), *I*^2^ = 0, *P* for effect = 0.251] (Figure 8).

**Figure 9:**
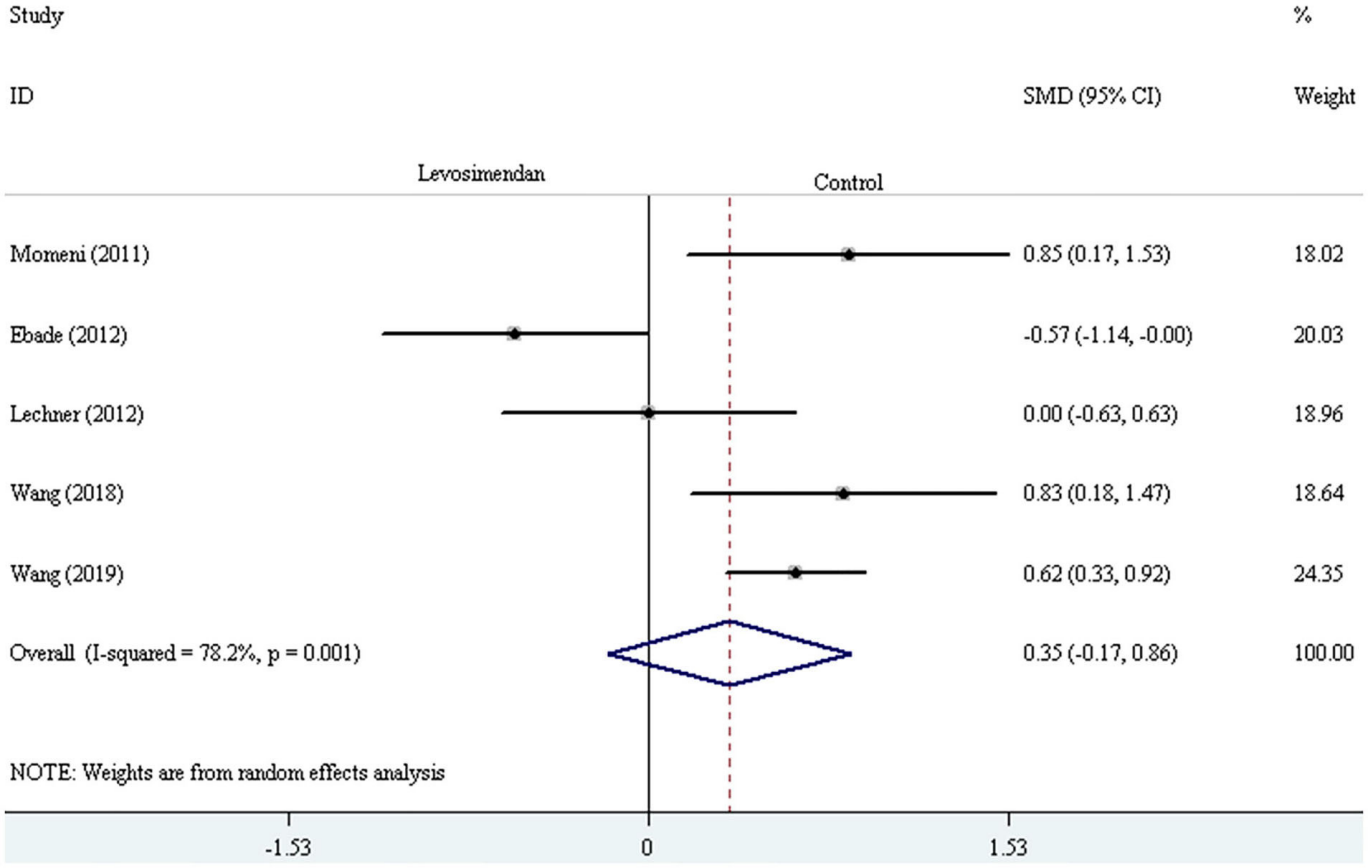
The comparison of the duration of mechanical ventilation (or intubation) between levosimendan and control drugs or placebo.

**Figure 10:**
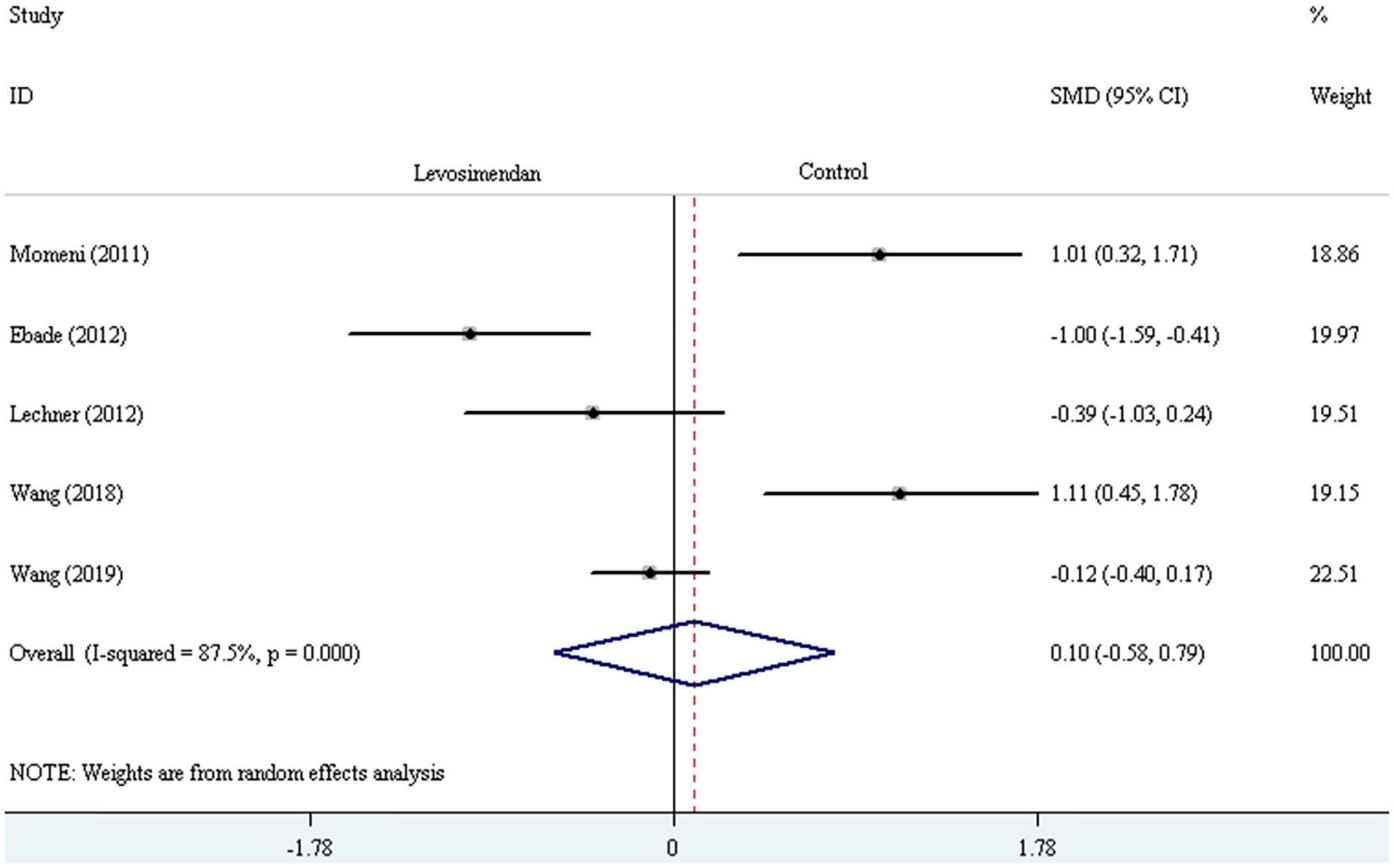
The comparison of the duration of ICU stay between levosimendan and control drugs or placebo.

Five trials reported the duration of mechanical ventilation (or intubation) and ICU stay (24–26, 29, 30). The random-effect model with SMD was selected to evaluate the duration of mechanical ventilation (or intubation) and ICU stay, and the pooled results did not demonstrate a significant difference comparing levosimendan with control drugs (and placebo) [mechanical ventilation (or intubation) time: SMD = 0.35, 95% CI (−0.17, 0.86), *I*^2^ = 78.2%, *P* for effect = 0.188; ICU stay time: SMD = 0.16, 95% CI (−0.46, 0.78), *I*^2^ =84.8%, *P* for effect = 0.620] (Figures 9, 10).

## Discussion

This meta-analysis included 6 RCTs and 1 CCT that compared the prophylactic effect of levosimendan vs. placebo or other inotropes on all-cause mortality and morbidity in pediatric patients undergoing cardiac surgery. The pooled results showed that perioperative levosimendan administration did not attenuate the all-cause mortality, AKI incidence and shorten mechanical ventilation and ICU stay time but strikingly reduced LCOS incidence in children following cardiac surgery compared with other inotropes and placebo by analyzing the included literatures.

The rates of mortality and morbidity are high in children undergoing congenital heart surgery. According to an observational cohort study, the mortality rate was about 5% in pediatric patients undergoing cardiac surgery during a 10-year follow-up and was much higher than that of the group of children without CHD and not undergoing surgery (0.1%) (31). The patients with younger age (<1 years) suffered higher 30-day mortality following cardiac surgery than those with older age (32, 33). In addition, the higher incidence of AKI and end-stage kidney disease (ESKD) was also exhibited in children following congenital heart surgery (31, 34). Furthermore, AKI, renal replacement therapy, and prolonged mechanical ventilation could significantly increase in-hospital and long-term mortality (35–37). Therefore, reducing mortality is a focus of clinical attention in children undergoing cardiac surgery.

LCOS can cause many fatal complications including AKI, ESKD, and even death in children undergoing open heart cardiac surgery for CHD, and younger age, preoperative cardiac dysfunction, and CPB are regarded as the considerable pathogenic factors of LCOS (4, 6, 38, 39). Perioperative sustained stable hemodynamics can significantly ameliorate reduced cardiac function following cardiac surgery (40). Inotrope administration in the perioperative period is the main method to maintain hemodynamic stability in current clinical practice. Levosimendan is also called inodilator which has both inotropic and lusotropic effects on the myocardium (41). Levosimendan exerts its effects through two main mechanisms of action: (1) increasing sensitivity of Ca^2+^ to cardiomyocyte by binding troponin C and (2) opening of the K^+^-dependent channels in vascular smooth muscle cells: which produce the effects of positive inotropy, lusotropy, vasodilation (systemic, pulmonary, and coronary), and cardioprotection (13–15). Many studies have reported the perioperative utility of levosimendan for preventing and treating LCOS in adult patients undergoing cardiac surgery. However, prophylactic levosimendan only demonstrated superiority in reducing LCOS incidence, mortality, and AKI incidence in patients with moderate and low ejection fraction compared with placebo (20, 42, 43). Considering that levosimendan is not a routine drug in children and high cost, there are a limited number of clinical studies with small sample size about perioperative levosimendan administration in pediatric patients undergoing cardiac surgery.

The meta-analysis involving 5 trials from Hummel et al. primarily investigated the effect of prophylactic levosimendan on mortality and LCOS incidence in pediatric patients undergoing surgery for congenital heart disease (9). Moreover, they did not draw a robust conclusion due to low quality of evidence. We enrolled another two latest papers, and the current data were still insufficient to make any definitive conclusions. As the number of high-quality studies in pediatric patients using levosimendan increases, the exact conclusions will be obtained.

We conducted subgroup analyses according to study designs, control drugs, time of study drug infusion onset, and duration of study drug infusion to investigate whether these subgroups could produce significant difference in all-cause mortality between groups of levosimendan and control. However, we did not obtain a significant difference in any one of the subgroups. Considering that none of the included trials regarded mortality as the primary endpoint, the quality of evidence of every trial was low, and the pooled result was questionable. We did not perform sensitivity analysis because there was no heterogenicity in primary outcome among these included trials (*I*^2^ = 0). Regarding the secondary outcomes, high heterogenicity was found in time of mechanical ventilation (*I*^2^ = 78.2%) and ICU stay (*I*^2^ = 84.8%), and we deemed that high heterogenicity may result from diversified factors, like different study design, drug administration method, time units, and perioperative management ideas. The random-effect model was used to analyze the two results, because it can decrease the effect of significant heterogeneity on the results, although this method does not solve heterogeneity (44, 45). As a result, we obtained a significant difference in LCOS incidence but did not find any statistical difference in AKI incidence, and time of mechanical ventilation and ICU stay between groups of levosimendan and control.

We should elaborate the strengths and limitations of this meta-analysis. First, this meta-analysis presented a comprehensive and up-to-date analysis of levosimendan vs. other inotropic agents and placebo in pediatric patients. Second, this meta-analysis indicated that more high-quality trials with large number of participants were required to investigate the effect of perioperative utility of levosimendan on post-operative complications in pediatric patients undergoing cardiac surgery. However, the high cost of levosimendan may be the biggest obstacle conducting a large sample-size trial. Third, we assessed the mortality risk of included trials according to STS-EACTS or RACHS, which attenuated the effect of imbalanced severity of conditions on post-operative mortality and morbidity. Some limitations should be taken into account in this meta-analysis. Foremost, the mortality was not the primary outcome of every included trials; thus, the pooled result may be unreliable due to mismatched sample size. In addition, all included trials were conducted as single center and with limited sample size, which elevated the risk of unreliable results. Thirdly, most of the included trials were assessed to be high-risk bias, and these trials with bias may affect the authenticity of pooled results. Lastly, the mortality was reported during different follow-up times, thereby leading to unreliable pooled results of this meta-analysis. However, considering this meta-analysis enrolled the maximum number of relevant studies, the results were most convincing.

## Conclusions

Compared with other inotropes and placebo, perioperative administration of levosimendan did not decrease the rates of mortality and AKI and shorten the time of mechanical ventilation (or intubation) and ICU stay in pediatric patients undergoing open heart cardiac surgery under CPB. However, levosimendan infusion was associated with reduced LCOS incidence through pooling the data comparing levosimendan with milrinone, standard inotropic management, and placebo. Because a limited number of trials with a small sample size reported the levosimendan-related mortality in pediatric patients undergoing corrective surgery for CHD and the primary outcome of every enrolled trial was not mortality, the current data were insufficient to make the conclusions. Therefore, high-quality RCTs with large number of patients were required to further investigate the effect of prophylactic levosimendan on all-cause mortality in pediatric patients undergoing corrective surgery for CHD.

## Data Availability Statement

The datasets presented in this study can be found in online repositories. The names of the repository/repositories and accession number(s) can be found in the article/Supplementary Material.

## Author Contributions

HW and LZ designed the meta-analysis and independently evaluated the quality of included articles. LZ, FY, and XW supervised the acquisition and analysis of the data. HW and YL were independently responsible for reviewing the titles, abstracts, or both and summarized the data of the included literatures. QL conducted the statistical analysis of data. HW wrote the manuscript. All authors contributed to the article and approved the submitted version.

## Conflict of Interest

The authors declare that the research was conducted in the absence of any commercial or financial relationships that could be construed as a potential conflict of interest.

## Supplementary Material

Supplementary Figure 1 Risk of bias graph: review authors' judgements about each risk of bias item presented as percentages across all included studies.

Supplementary Figure 2 Risk of bias summary: review authors' judgements about each risk of bias item for each included study.

Supplementary Table 1 The guidelines of the 2009 PRISMA (Preferred Reporting Items for Systematic reviews and Meta-analyses).

Supplementary Table 2 The Newcastle-Otawa Quality Assessment Scale (NOS).

Supplementary Table 3 Procedure names, STS-EACTS mortality categories, and scores of six included trials.

Supplementary Table 4 The statistical result of STS-EACTS scores in included trials between the groups of levosimendan and control.
