# Supplementary figures and images for: Effect of Prophylactic Levosimendan on All-Cause Mortality in Pediatric Patients Undergoing Cardiac Surgery—An Updated Systematic Review and Meta-Analysis
Source: Front Pediatr. 2020 Aug 14;8:456. doi: 10.3389/fped.2020.00456 (PMC7456871; doi:10.3389/fped.2020.00456)

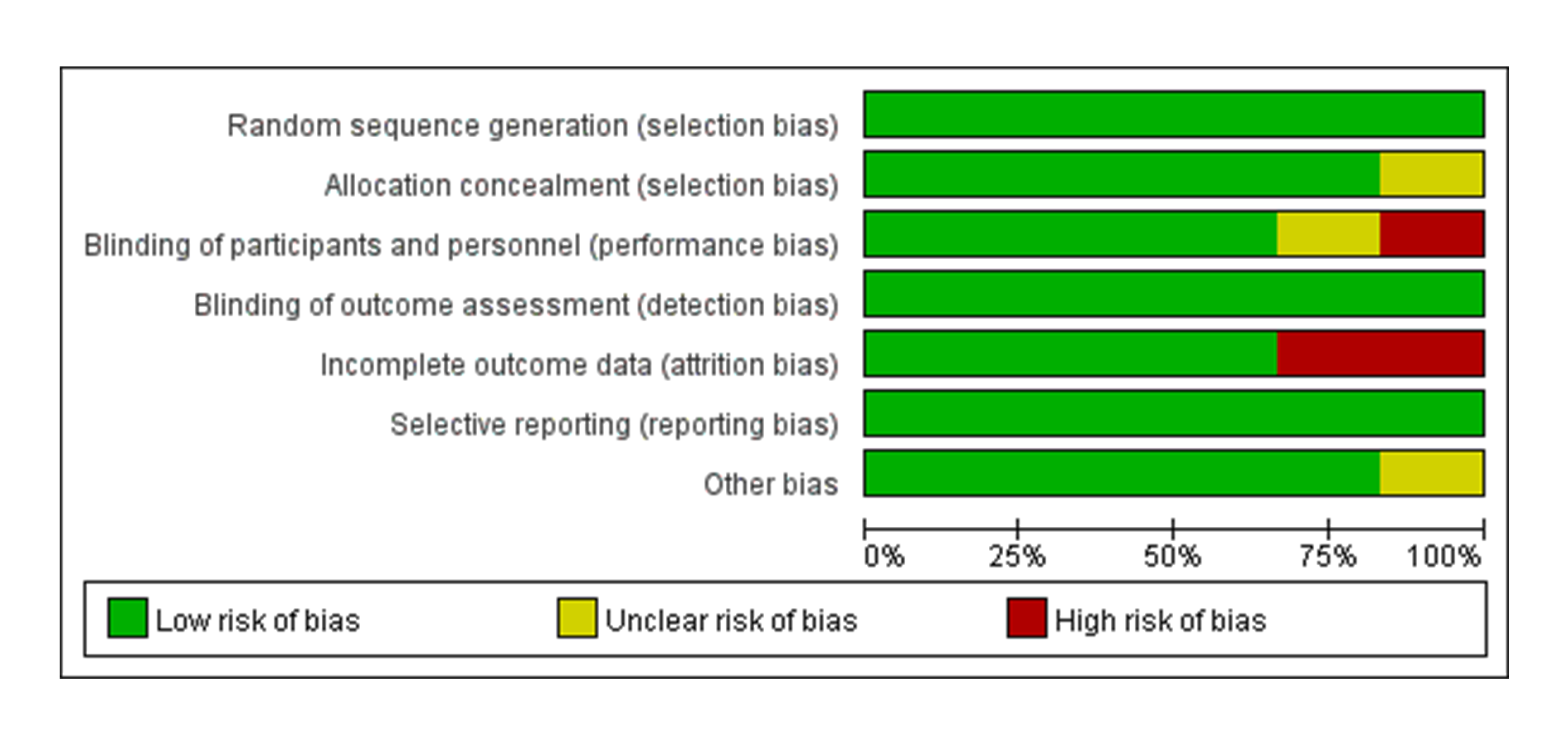

Supplement: Supplementary Figure 1 — Risk of bias graph: review authors' judgements about each risk of bias item presented as percentages across all included studies. [file Image_1.TIF]

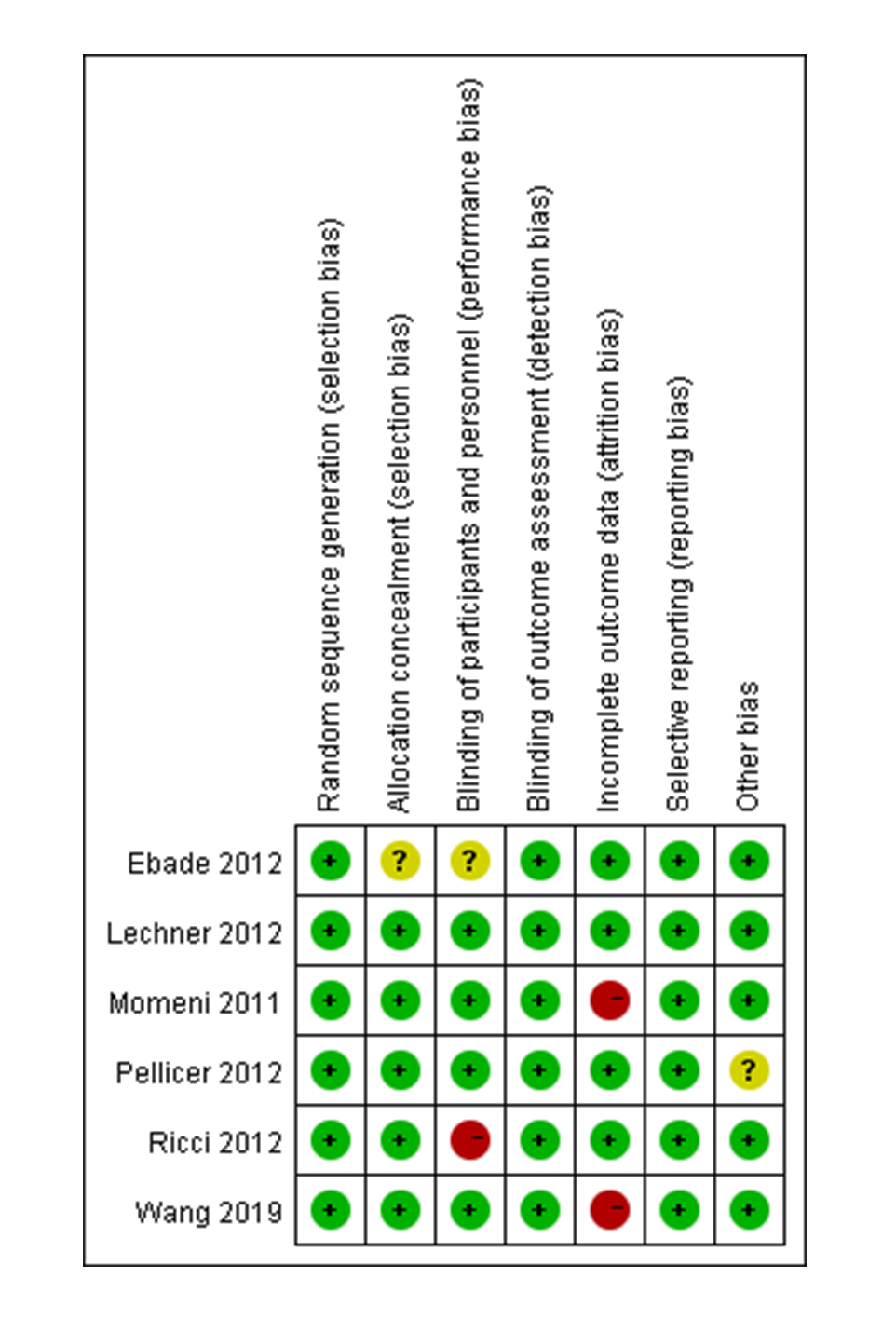

Supplement: Supplementary Figure 2 — Risk of bias summary: review authors' judgements about each risk of bias item for each included study. [file Image_2.TIF]
