# Supplementary material for: Effect of Prophylactic Levosimendan on All-Cause Mortality in Pediatric Patients Undergoing Cardiac Surgery—An Updated Systematic Review and Meta-Analysis
Source: Front Pediatr. 2020 Aug 14;8:456. doi: 10.3389/fped.2020.00456 (PMC7456871; doi:10.3389/fped.2020.00456)
Supplement: Supplementary Table 1 — The guidelines of the 2009 PRISMA (Preferred Reporting Items for Systematic reviews and Meta-analyses). [file Table_1.DOC]

| **Section/topic** | **#** | **Checklist item** | **Reported on page #** |
| --- | --- | --- | --- |
| **TITLE** | | |  |
| Title | 1 | Effect of levosimendan on all-cause mortality in pediatric patients undergoing cardiac surgery- An update systematic review and meta-analysis | P 1 |
| **ABSTRACT** | | |  |
| Structured summary | 2 | **Background:** Low cardiac output syndrome (LCOS) remains a serious problem, and contributes to substantial morbidity and mortality in pediatric patients undergoing cardiac surgery. Levosimendan, a calcium sensitizer, enhances the myocardial function by generating more energy-efficient myocardial contractility than achieved through adrenergic stimulation with catecholamines. We conducted this meta-analysis to primarily investigate the effects of levosimendan on all-cause mortality in pediatric patients undergoing cardiac surgery under cardiopulmonary bypass.  **Methods:** The databases of Pubmed, Embase and Cochrane Library were searched till 21st March 2020. The eligible criteria were participants with age<18 yr and undergoing cardiac surgery for congenital heart disease (CHD), and studies of comparison between levosimendan and placebo or other inotropes. Stata version 12.0 was used to perform statistical analyses.  **Results:** 6 Randomized Controlled Trials (RCTs) and 1 Case-Control Trial (CCT) including 436 patients were included. The results showed that levosimendan did not significantly decrease all-cause mortality compared with control drugs (and placebo) in children undergoing cardiac surgery [RR=0.66, 95% CI (0.25, 1.74), I^2^=0, *P* for effect=0.403]. Furthermore, perioperative levosimendan administration did not significantly reduced AKI incidence [RR=0.60, 95% CI (0.25, 1.44), I^2^=0, *P* for effect=0.251], and shortened mechanical ventilation and ICU stay time compared with other inotropes and placebo by analyzing the included literatures [mechanical ventilation (or intubation) time: SMD=0.35, 95% CI (-0.17, 0.86), I^2^=78.2%, *P* for effect=0.188; ICU stay time: SMD=0.16, 95% CI (-0.46, 0.78), I^2^=84.8%, *P* for effect=0.620].  **Conclusion:** Compared with other inotropes and placebo, perioperative administration of levosimendan did not exhibit its superiority in decreasing rates of mortality and AKI and shortening the time of mechanical ventilation (or intubation) and ICU stay after corrective surgery in pediatric patients for CHD.  **Keywords:** Low cardiac output syndrome, levosimendan, pediatrics, cardiac surgery, mortality | P 2 |
| **INTRODUCTION** | | |  |
| Rationale | 3 | Low cardiac output syndrome (LCOS) refers to the clinical manifestation of mismatched oxygen supply and demand due to cardiovascular dysfunction following cardiac surgery. The diagnostic criteria of LCOS include cardiac index (CI)<2.0 L/min/m, a systolic blood pressure <90 mmHg, signs of tissue hypoperfusion (cold periphery, clammy skin, confusion, oliguria, elevated lactate level) in the absence of hypovolemia, and inotropic agents or mechanical circulatory support to improve patient’s hemodynamics. LCOS often occurs during 9-12 h after cardiopulmonary bypass (CPB). The incidence of LCOS is nearly 10% (9.98%) in children (0-18 years old) after corrective surgery for congenital heart disease (CHD), while in neonates and infants as high as 25%-65%. The development of LCOS is highly associated with acute kidney injury (AKI), prolonged time of mechanical ventilation and ICU stay, and even higher mortality.  Some inotropic agents has been widely used in clinical practice to prevent and treat LCOS. Catecholamines (epinephrine, norepinephrine, dopamine and dobutamine) and phosphodiesterase inhibitor (milrinone) are the traditional prophylactic and therapeutic medications. But these drugs are associated with considerable side effects. Levosimendan is a novel inotropic drug that enhances myocardial contractility through increasing sensitivity of calcium ion to cardiomyocytes. In addition, levosimendan also has the pharmacological feature of dilatation of blood vessels (systemic, pulmonary and coronary) due to its role of K^+^ efflux, thereby decreasing cardiac pre-and afterload. Therefore, levosimendan elevates cardiac contractility, meanwhile does not increase cardiac oxygen consumption. Besides, levosimendan has pharmacological feature of myocardial preservation as well. Therefore, levosimendan has a theoretical advantage in improving postoperative cardiac function and reducing postoperative complications and mortality in pediatric patients undergoing cardiac surgery. | P 3 |
| Objectives | 4 | We designed this meta-analysis to primarily observe the effect of perioperative levosimendan administration on all-cause mortality in pediatric patients following cardiac surgery under CPB. | P 3 |
| **METHODS** | | |  |
| Protocol and registration | 5 | No registration |  |
| Eligibility criteria | 6 | The inclusion criteria included: 1) participants with age<18 years; 2) management with prophylactic levosimendan and placebo or other inotropic agents. The exclusion criteria included: 1) participants with age≥18 years; 2) review or meta-analysis; 3) basic research; 4) article published as abstract, letter, case report, editorial, note, method or protocol; 5) article presented in non-English language. | P 4 |
| Information sources | 7 | We searched the databases including "Pubmed", "Embase" and "Cochrane Library" through PICOS (Population, Intervention, Comparison, Outcome, Study design) method by the time to 21st March 2020. | P 4 |
| Search | 8 | The entry words included “infant” or “newborn” or “child” or “children” or “pediatrics” or “neonate” and “simendan” or “levosimendan” and “ thoracic surgery” or “surgery, thoracic” or “surgery, cardiac” or “cardiac surgery” or “heart surgery” and “mortality” or “mortalities” or “case fatality rate” or “rate, case fatality” or “rates, fatality” or “death rate” or “rate, death” or “rates death” or “mortality rates”, and the search scope was "all fields". Because all studies about effect of levosimendan versus placebo or other inotropic drugs on mortality in pediatric patients were eligible in this meta-analysis, we did not confine the search words of control drugs and study design. |  |
| Study selection | 9 | The inclusion criteria included: 1) participants with age<18 yr; 2) management with prophylactic dexmedetomidine and placebo or other sedatives. The exclusion criteria included: 1) participants with age≥18 yr; 2) management with dexmedetomidine alone; 3) review or meta-analysis; 4) basic research; 5) article published as abstract, letter, case report, editorial, note, method or protocol; 6) article presented in non-English language. | P 4 |
| Data collection process | 10 | Two authors were independently responsible for reviewing the titles, abstracts or both and summarized the data of the included literatures. Another three authors were in charge of the data discrepancy adjustment. | P 4-5 |
| Data items | 11 | 1) authors; 2) publication year; 3) number of the total participants in each study; 4) age range of all the participants; 5) country of publication; 6) time of levosimendan or other drugs administration; 7) infusion speed of levosimendan or other sedatives; 8) number of patients suffering death or acute kidney injury (AKI), and time of mechanical ventilation (or intubation) and ICU stay following cardiac surgery. | P 4 |
| Risk of bias in individual studies | 12 | The Cochrane Collaboration Risk of Bias Assessment tool was used to assess the risk of bias of all included RCTs, and the Newcastle-Otawa Quality Assessment Scale (NOS) was used to assess the bias risk of case-control trials (CCTs) were assessed by two authors independently. If the two authors had the different assessment results, they consulted the third or the forth one. Eventually, we reached consensus. | P5 |
| Summary measures | 13 | The dichotomous outcomes were reported as relative risk (RR) with 95% confidence interval (CI). Because the different time units (hours and days) were presented in mechanical ventilation (or intubation) time and ICU stay time, the two continuous outcomes were analyzed as standard mean difference (SMD) with 95% CI.The statistical tests were two-sided and *P* value for overall effect <0.05 was considered significant differences. | P 5 |
| Synthesis of results | 14 | The values of I^2^ and the Mantel-Haenszel chi-square test (*P* value for heterogeneity) were used to evaluate the heterogeneity of included studies. And the values of I^2^<40%, 40%-60%, and >60% represented low, moderate and high heterogeneity, respectively. A *P* value for heterogeneity<0.1 or I^2^ >50% was regarded as high heterogeneity and the method of random-effect model analysis was applied to pool the data. | P 5 |

Page 1 of 2

| **Section/topic** | **#** | **Checklist item** | **Reported on page #** |
| --- | --- | --- | --- |
| Risk of bias across studies | 15 | Bias risk of 6 RCTs was assessed by the Cochrane Collaboration Risk of Bias Assessment tool. Random sequence generation was assessed as a low risk of bias in 6 studies (100%), allocation concealment in 5 studies (83%), blinding of participants in 4 studies (67%), blinding of outcome assessment in 6 studies (100%), incomplete outcome data in 4 studies (67%), selective outcome reporting in 6 studies (100%), and other bias in 5 studies (Supplementary Fig 1 and 2). The CCT study obtained 7 stars through NOS. 1 RCT^23^ and CCT^27^ were assessed to be high quality. | P 6-7 |
| Additional analyses | 16 | Subgroup analyses were conducted for primary outcome according to study designs, control drugs, onset time of study drugs, and duration of study drug infusion. | P 5-6 |
| **RESULTS** | | |  |
| Study selection | 17 | See Fig. 1 | P 5-6 |
| Study characteristics | 18 | For each study, present characteristics for which data were extracted (e.g., study size, PICOS, follow-up period) and provide the citations. | P 6 |
| Risk of bias within studies | 19 | Present data on risk of bias of each study and, if available, any outcome level assessment (see item 15). | P 6-7 |
| Results of individual studies | 20 | For all outcomes considered (benefits or harms), present, for each study: (a) simple summary data for each intervention group (b) effect estimates and confidence intervals, ideally with a forest plot. | P 7-8 |
| Synthesis of results | 21 | The fixed-effect model with RR was selected to evaluate the primary outcome and the pooled result did not demonstrate significant difference in all-cause mortality compared levosimendan with control drugs (and placebo) [RR=0.66, 95% CI (0.25, 1.74), I^2^=0, *P* for effect=0.403].  We conducted the subgroup analyses according to study designs, control drugs, time of study drug infusion onset, and duration of study drug infusion. There was no significant difference in all-cause mortality between groups of levosimendan and control according to study designs [RCTs: RR=0.51, 95% CI (0.17, 1.55), *P* for effect=0.233; CCT: RR=2.00, 95% CI (0.20, 20.33), *P* for effect=0.558], control drugs [milrinone: RR=0.28, 95% CI (0.01, 6.10), *P* for effect=0.416; standard inotropic mamagement: RR=0.74, 95% CI (0.17, 3.14), *P* for effect=0.680; placebo: RR=0.74, 95% CI (0.17, 3.22), *P* for effect=0.691] (Fig 4), time of drug infusion onset [before surgery: RR=0.28, 95% CI (0.01, 6.10), *P* for effect=0.416; during surgery: RR=0.32, 95% CI (0.04, 2.94), *P* for effect=0.316; after surgery: RR=0.99, 95% CI (0.30, 3.33), *P* for effect=0.990], and duration of study drug infusion [24h after surgery: RR=2.00, 95% CI (0.20, 20.33), *P* for effect=0.558; 48h after starting infusion: RR=0.61, 95% CI (0.17, 2.24), *P* for effect=0.454; 72 h after starting infusion: RR=0.32, 95% CI (0.04, 2.94), *P* for effect=0.316).  4 trials reported the incidence of AKI. The pooled result did not demonstrate significant difference in AKI incidence compared levosimendan with control drugs (and placebo) through the method of fixed-effect model with RR [RR=0.60, 95% CI (0.25, 1.44), I^2^=0, *P* for effect=0.251].  5 trials reported the duration of mechanical ventilation (or intubation) and ICU stay. The random-effect model with SMD was selected to evaluate the duration of mechanical ventilation (or intubation) and ICU stay and the pooled results did not demonstrate significant difference compared levosimendan with control drugs (and placebo) [mechanical ventilation (or intubation) time: SMD=0.35, 95% CI (-0.17, 0.86), I^2^=78.2%, *P* for effect=0.188; ICU stay time: SMD=0.16, 95% CI (-0.46, 0.78), I^2^=84.8%, *P* for effect=0.620] | P 7-8 |
| Risk of bias across studies | 22 | Present results of any assessment of risk of bias across studies (see Item 15). | P 6-7 |
| Additional analysis | 23 | Give results of additional analyses, if done (e.g., sensitivity or subgroup analyses, meta-regression [see Item 16]). | P 8 |
| **DISCUSSION** | | |  |
| Summary of evidence | 24 | This meta-analysis included 6 RCTs and 1 CCT that compared the prophylactic effect of levosimendan versus placebo or other inotropes on all-cause mortality in pediatric patients undergoing cardiac surgery. The result showed that perioperative levosimendan administration did not decrease the all-cause mortality after cardiac surgery in pediatric patients compared with other inotropes and saline, respectively. Furthermore, perioperative levosimendan administration did not exhibit its superiority in reducing AKI incidence, and shortening mechanical ventilation and ICU stay time in children following cardiac surgery compared with other inotropes and placebo by analyzing the included literatures. | P 8 |
| Limitations | 25 | Foremost, the mortality was not the primary outcome of every included trials, thus, the pooled result may be unreliable due to mismatched sample size. In addition, most of included trials were assessed to be high-risk bias, and these trials with bias may affect the authenticity of pooled results. Lastly, the mortality was reported during different follow-up time, thereby leading to unreliable pooled results of this meta-analysis. | P 10-11 |
| Conclusions | 26 | Compared with other inotropes and placebo, perioperative administration of levosimendan did not exhibit its superiority in decreasing rates of mortality and AKI and shortening the time of mechanical ventilation (or intubation) and ICU stay in pediatric patients undergoing open heart cardiac surgery under CPB. But because a limited number of trials with small sample size reported the levosimendan related mortality in pediatric patients undergoing corrective surgery for CHD and the primary outcome of these trials was not mortality, the pooled result of this meta-analysis was unreliable. Therefore, high-quality RCTs with large number of patients were required to further investigate the effect of prophylactic levosimendan on all-cause mortality in pediatric patients undergoing corrective surgery for CHD. | P 11 |
| **FUNDING** | | |  |
| Funding | 27 | No |  |

*From:*  Moher D, Liberati A, Tetzlaff J, Altman DG, The PRISMA Group (2009). Preferred Reporting Items for Systematic Reviews and Meta-Analyses: The PRISMA Statement. PLoS Med 6(7): e1000097. doi:10.1371/journal.pmed1000097

For more information, visit: **www.prisma-statement.org**.

Page 2 of 2
