# Supplementary material for: Effect of Prophylactic Levosimendan on All-Cause Mortality in Pediatric Patients Undergoing Cardiac Surgery—An Updated Systematic Review and Meta-Analysis
Source: Front Pediatr. 2020 Aug 14;8:456. doi: 10.3389/fped.2020.00456 (PMC7456871; doi:10.3389/fped.2020.00456)
Supplement: Supplementary Table 3 — Procedure names, STS-EACTS mortality categories, and scores of six included trials. [file Table_3.DOC]

Supplementary table 3. Procedure names, STS-EACTS mortality categories and scores of six included trials

| **Author/Publication year** | **Levosimendan** | | | | **Control** | | | |
| --- | --- | --- | --- | --- | --- | --- | --- | --- |
|  | **Procedures** | **No.of patients** | **Category** | **Score** | **Procedures** | **No.of patients** | **Category** | **Score** |
| Momeni 2011 | Arterial switch repair | 4 | 3 | 1.7 | CAVC + DA closure | 1 | 2+2 | 2.4 |
|  | ALCAPA | 1 | 3 | 1.6 | VSD closure | 7 | 1 | 0.6 |
|  | Arterial switch repair + VSD closure | 1 | 4 | 2.8 | CAVC | 1 | 2 | 1.3 |
|  | CAVC | 2 | 2 | 1.3 | Truncus arteriosus  repair | 3 | 4 | 3.4 |
|  | VSD closure + DA  closure | 1 | 1+2 | 1.7 | Arterial switch repair | 3 | 3 | 1.7 |
|  | VSD closure | 2 | 1 | 0.6 | Multiple VSD closures +  ASD closure | 1 | 2+1 | 1.6 |
|  | Senning Rastelli repair | 1 | 3+4 | 4.5 | Multiple VSD closures | 1 | 2 | 1.5 |
|  | TAPVR | 1 | 4 | 2.6 | Mitral repair + VSD closure | 1 | 3+1 | 2.7 |
|  | Truncus arteriosus  repair | 3 | 4 | 3.4 |  |  |  |  |
|  | VSD closure + ASD  closure | 1 | 2 | 1.3 |  |  |  |  |
|  | DORV repair + VSD  closure | 1 | 3+1 | 2.6 |  |  |  |  |

Continued

| **Author/Publication year** | **Levosimendan** | | | | **Control** | | | |
| --- | --- | --- | --- | --- | --- | --- | --- | --- |
|  | **Procedures** | **No.of patients** | **Category** | **Score** | **Procedures** | **No.of patients** | **Category** | **Score** |
| Ebade 2012 | ASD or VSD | 25 | 1 | 0.2 | ASD or VSD | 25 | 1 | 0.2 |
| Lechner 2012 | VSD closure | 7 | 1 | 0.6 | VSD closure | 3 | 1 | 0.6 |
|  | CAVC | 2 | 2 | 1.3 | CAVC | 5 | 2 | 1.3 |
|  | Multiple VSD closures | 1 | 2 | 1.5 | TAPVC | 1 | 4 | 2.6 |
|  | Extended coarctation repair | 1 | 2 | 0.9 | Arterial switch repair | 6 | 3 | 1.7 |
|  | Arterial switch repair | 6 | 3 | 1.7 | Arterial switch repair+ VSD closure | 5 | 4 | 2.8 |
|  | Arterial switch repair + VSD closure | 2 | 4 | 2.8 |  |  |  |  |
| Pellicer 2012 | D-TGA | 4 | 4 | 2.5 | D-TGA | 4 | 4 | 2.5 |
|  | D-TGA+VSD | 3 | 4+1 | 3.1 | D-TGA+VSD | 1 | 4+1 | 3.1 |
|  | AoCo+AoArHypo+ASD | 1 | 3+3+1 | 3.9 | D-TGA+ASD | 1 | 4+1 | 2.6 |
|  | TAPVR | 1 | 4 | 2.6 | AoCo+ASD+VSD | 1 | 3+1 | 2.1 |
|  | T. Fallot | 1 | 1 | 0.7 | AoCo+HypoAsAo+AoArHypo+  VSD+ASD | 1 | 3+3 | 4.2 |
|  | AoArHypo+VSD+ASD | 1 | 2+1+1 | 2.2 | AoArHypo+VSD+ASD | 1 | 2+1+1 | 2.2 |
| Ricci 2012 | Aortic arch repair | 3 | 2 | 2.2 | Aortic arch repair | 2 | 3 | 2.2 |
|  | ALCAPA | 1 | 3 | 1.6 | Interruption of  aortic arch | 1 | 4 | 3.5 |

Continued

| **Author/Publication year** | **Levosimendan** | | | | **Control** | | | |
| --- | --- | --- | --- | --- | --- | --- | --- | --- |
|  | **Procedures** | **No.of patients** | **Category** | **Score** | **Procedures** | **No.of patients** | **Category** | **Score** |
|  | Interruption of  aortic arch | 2 | 4 | 3.5 | D-TGA+VSD | 4 | 4+1 | 3.1 |
|  | D-TGA+VSD | 5 | 4+1 | 3.1 | D-TGA | 20 | 4 | 2.5 |
|  | D-TGA | 17 | 4 | 2.5 | D-TGA +aortic coarctation | 1 | 4+1 | 2.9 |
|  | D-TGA +aortic coarctation | 2 | 4+1 | 2.9 | Truncus arteriosus | 3 | 4 | 3.4 |
|  | Truncus arteriosus | 2 | 4 | 3.4 |  |  |  |  |
| Wang 2018 | ALCAPA | 20 | 3 | 1.6 | ALCAPA | 20 | 3 | 1.6 |

STS-EACTS: The Society of Thoracic Surgeons-European Association for Cardiothoracic Surgery; ALCAPA: anomalous left coronary artery from the pulmonary artery; CAVC: complete atrioventricular canal; DA: ductus arteriosus; ASD: atrial septal defect; VSD: ventricular septal defect; TAPVC: total anomalous pulmonary venous connection; TAPVR: total anomalous pulmonary venous return; DORV: double-outlet right ventricle; D-TGA: right transposition of great arteries; AoCo: aortic coarctation; AoArHypo: aortic arch hypoplasia; HypoAsAo: ascending aorta hypoplasia; T. Fallot: tetralogy of Fallot
