# Supplementary material for: Effect of Prophylactic Levosimendan on All-Cause Mortality in Pediatric Patients Undergoing Cardiac Surgery—An Updated Systematic Review and Meta-Analysis
Source: Front Pediatr. 2020 Aug 14;8:456. doi: 10.3389/fped.2020.00456 (PMC7456871; doi:10.3389/fped.2020.00456)
Supplement: Supplementary Table 4 — The statistical result of STS-EACTS scores in included trials between the groups of levosimendan and control. [file Table_4.DOC]

|  | **No. Of patients** | **Mean±SD** | ***p* value** |
| --- | --- | --- | --- |
| Levosimendan versus Control | 125 versus 123 | 1.715±1.072 versus 1.697±1.044 | 0.773 |

Supplementary table 4. The statistical result of STS-EACTS scores in included trials between the groups of levosimendan and control
